# Antimicrobial Activity of Pantothenol against Staphylococci Possessing a Prokaryotic Type II Pantothenate Kinase

**DOI:** 10.1264/jsme2.ME13178

**Published:** 2014-04-22

**Authors:** Shigeru Chohnan, Misa Murase, Kota Kurikawa, Kodai Higashi, Yuta Ogata

**Affiliations:** 1 Department of Bioresource Science, Ibaraki University College of Agriculture, 3–21–1 Chuo, Ami, Ibaraki 300–0393, Japan; 2 Department of Applied Life Science, United Graduate School of Agricultural Science, Tokyo University of Agriculture and Technology, 3–5–8 Saiwai, Fuchu, Tokyo 183–8509, Japan

**Keywords:** staphylococci, pantothenol, pantothenate kinase, coenzyme A, antimicrobial activity

## Abstract

Pantothenol is a provitamin of pantothenic acid (vitamin B_5_) that is widely used in healthcare and cosmetic products. This analog of pantothenate has been shown to markedly inhibit the phosphorylation activity of the prokaryotic type II pantothenate kinase of *Staphylococcus aureus*, which catalyzes the first step of the coenzyme A biosynthetic pathway. Since type II enzymes are found exclusively in staphylococci, pantothenol suppresses the growth of *S. aureus*, *S. epidermidis*, and *S. saprophyticus*, which inhabit the skin of humans. Therefore, the addition of this provitamin to ointment and skincare products may be highly effective in preventing infections by opportunistic pathogens.

Coenzyme A (CoA) functions as an acyl carrier and is an indispensable cofactor for all living cells. CoA is synthesized from pantothenate (vitamin B_5_), cysteine, and ATP through five enzymatic steps: pantothenate kinase (CoaA in prokaryotes and PanK in eukaryotes), phosphopantothenoylcysteine synthetase (CoaB), phosphopantothenoylcysteine decarboxylase (CoaC), phosphopantetheine adenyltransferase (CoaD), and dephospho-CoA kinase (CoaE) (8). The pantothenate kinases that catalyze the phosphorylation of pantothenate are key enzymes in the CoA biosynthetic pathway, and have been divided into four groups based on their amino acid sequences, *i.e.*, prokaryotic type I, II, and III CoaAs and eukaryotic PanK (12). Prokaryotic type I CoaA and PanK are known to be sensitive to CoASH (nonesterified CoA) and acyl-CoAs (12, 19), whereas type II and III CoaAs are resistant to the end-products of the pathway (2, 11). In addition, the type III CoaA requires monovalent cations, *i.e.*, K^+^ or NH_4_^+^, for its enzymatic activity (7). Thus, diversity exists amongst the structures and properties of pantothenate kinases, and these essential enzymes have become attractive drug targets for the development of novel antimicrobial agents (5, 13, 17). N-substituted pantothenamides (*N*-pentylpantothenamide and *N*-heptylpantothenamide) and CJ-15,801 produced by *Seimatosporium* sp., which have the ability to inhibit the prokaryotic type II CoaA, have been shown to effectively interfere with the growth of *Staphylococcus aureus*, which uses the type II CoaA in its CoA biosynthetic pathway (4, 11, 18, 20, 21).

The antimicrobial activity of pantothenol, a provitamin of pantothenic acid, against lactic acid bacteria, which require pantothenic acid for their growth, was identified in the 1940s (Fig. 1A) (16). Pantothenol has recently been reported to suppress the phosphorylation activity of the prokaryotic type I CoaA from *Mycobacterium tuberculosis* as well as the proliferation of malaria by inhibiting parasite eukaryotic PanK(s) (10, 15). In the present study, the potential effectiveness of pantothenol as an antimicrobial agent was investigated.

The distribution of the homologous genes encoding the three types of CoaAs in bacteria was as follows: the type I CoaA, the genera *Corynebacterium*, *Lactobacillus*, *Lactococcus*, *Streptococcus*, *Rhizobium*, *Escherichia*, *Klebsiella*, *Salmonella*, *Serratia*, *Shigella*, *Yersinia*, *Coxiella*, *Shewanella*, *Haemophilus*, and *Vibrio*; the type II CoaA, the genus *Staphylococcus*; and the type III CoaA, the genera *Clostridium*, *Thermotoga*, *Thermus*, *Burkholderia*, *Neisseria*, *Campylobacter*, *Helicobacter*, *Francisella*, *Legionella*, *Pseudomonas*, and *Xanthomonas*. Thus, type I and III CoaAs are widely distributed while the type II CoaA is limited to staphylococci. Although the putative type II CoaA gene, together with the type III enzyme, is also conserved in some *Bacillus* species, the type II kinase of *B. anthracis* does not function *in vivo* (14). Therefore, the prokaryotic type I CoaA from *Escherichia coli* K-12 (*Ec*CoaA), the type II CoaA from *S. aureus* MW2 (*Sa*CoaA), and the type III CoaA from *Pseudomonas putida* JCM 20089 (*Pp*CoaA) were examined in this study. The expression plasmid for *Ec*CoaA, pET15b/bPanK, was obtained from Dr. Jackowski, St. Jude Children’s Research Hospital, USA (3). The *coaA* genes coding for *Sa*CoaA (MW2054) and *Pp*CoaA (accession number AB829254) were amplified by PCR, and the resulting DNA fragments were cloned into pET-28a(+) to generate pET-Sa-coaA and pET-Pp-coaA. *E. coli* BL21 (DE3) cells transformed with pET15b/bPanK, pET-Sa-coaA, or pET-Pp-coaA were grown in LB broth containing IPTG, and the recombinant enzymes were prepared using a nickel-chelating resin (Table S1 and Fig. S1). The inhibitory effect of pantothenol on pantothenate kinase activity was determined using d-[^14^C] pantothenate and ATP as substrates in the presence of d-pantothenol at concentrations of 0.5 to 10 mM (Fig. 1B).

Pantothenol reduced the activities of type I and II CoaAs, with a more potent effect being observed on the type II CoaA than on the type I CoaA, with an IC_50_ of 1.68 mM for the type II *Sa*CoaA. In the presence of 10 mM pantothenol, the activities of *Sa*CoaA and *Ec*CoaA were decreased to 14.5% and 42.1% of their maximal activities, respectively. Conversely, the type III CoaA (*Pp*CoaA) was not inhibited by pantothenol.

The pantothenol treatment significantly inhibited the phosphorylation activity of the prokaryotic type II CoaA. Although this effect was observed at a high IC_50_ in the millimolar range, humans have the ability to convert this provitamin to vitamin B_5_, pantothenic acid (1), and no apparent toxicity has been reported for pantothenol, even at oral dosage levels of 8 to 10 g daily (6). Hence, it is possible for pantothenol to act as an antimicrobial agent. The MIC values of pantothenol against bacteria possessing the prokaryotic type I, II, or III CoaA were determined by a broth-microdilution method. Bacto-tryptone (Becton, Dickinson, and Company) was employed as a medium for the estimation of bacterial growth, as its low pantothenic acid content (typically *ca*. 5.3 μg g^−1^) was unlikely to compete with pantothenol. The bacterial strains were grown to the mid-log phase in 1% (w/v) bacto-tryptone at 30°C, diluted in the same medium, and then added to medium containing 0 to 32 mM pantothenol at a density of 5 × 10^5^ colony-forming unit mL^−1^ in each well of a microplate. After 24 h of cultivation at 30°C, the turbidity at 600 nm was measured. There are many isolates, including hospital- and community-acquired methicillin-resistant *Staphylococcus aureus* among *S. aureus*, and the pantothenate kinase (*Sa*CoaA) from the strain MW2 shares almost the same amino acid sequence with the other strains of *S. aureus* (>99%-identity). Hence, *S. aureus* subsp. *aureus* type strain NBRC 100910 was used here instead of the MW2 strain. Furthermore, the strains of *S. epidermidis* NBRC 12993, *S. epidermidis* type strain NBRC 100911, and *S. saprophyticus* subsp. *saprophyticus* type strain NBRC 102446 were also examined. Although the amino acid sequence of the CoaA from *S. epidermidis* type strain NBRC 100911 was not available, the sequences of CoaAs from *S. epidermidis* NBRC 12993 and *S. saprophyticus* type strain NBRC 102446 showed 75%- and 68% identity to that from the MW2 strain. The growth of staphylococci possessing the prokaryotic type II CoaA was effectively suppressed by the addition of 32 mM pantothenol, showing 71.9% inhibition in *S. aureus* subsp. *aureus* NBRC 100910, 85.7% in *S. epidermidis* NBRC 12993, 98.0% in *S. epidermidis* NBRC 100911, and 99.7% in *S. saprophyticus* subsp. *saprophyticus* NBRC 102446 (Table 1). This result was consistent with the *in vitro* experiment using recombinant *Sa*CoaA (Fig. 1B). As shown in Table 1 and Fig. 2, the MIC values of *S. epidermidis* NBRC 100911 and *S. saprophyticus* subsp. *saprophyticus* NBRC 102446 were estimated to be 4 and 2 mM, respectively, although the growth of *S. aureus* subsp. *aureus* NBRC 100910 and *S. epidermidis* NBRC 12993 were not completely suppressed even in the presence of 32 mM. The inhibitory effect of pantothenol was abrogated by the addition of 1 μM pantothenate, and the IC_50_ values for *S. epidermidis* NBRC 100911 and *S. saprophyticus* NBRC 102446 shifted from 0.776 and 0.641 mM to 4.82 and 5.20 mM, respectively (Fig. 2C and D). This result clearly indicated that pantothenol also competed with pantothenate on the type II enzyme activities *in vivo*. On the other hand, pantothenol had little effect on the growth of *E. coli* and *P. putida* (Table 1), although the IC_50_ of the *E. coli* enzyme was calculated to be 6.06 mM (Fig. 1B). Thus, the inhibitory effect of pantothenol on bacterial growth depended on the types of CoaA that bacteria employed in their CoA biosynthetic pathways.

The expression of prokaryotic type II CoaA is known to be specific to the genus *Staphylococcus* (4), and pantothenol was found to be effective as a growth inhibitor of staphylococci using type II enzymes in this study. Since the inhibitory effect was reduced in the presence of a small amount of pantothenate (Fig. 2C and D), the oral administration of pantothenol may not have had a direct effect on bacterial growth because pantothenate, which is derived from food and produced by enterobacteria, is abundant in the body. However, since staphylococci including opportunistic pathogens such as *S. aureus*, *S. epidermidis*, and *S. saprophyticus*, which lead to impetigo, hospital-associated bloodstream infections, and urinary tract infection, populate the skin and mucous membranes of humans and other mammals, especially in moist areas such as the anterior nares, axillae, and perineal areas (9, 22), the use of ointment and skincare products containing pantothenol may be highly effective at preventing infections by staphylococci.

## Supplementary Information



## Figures and Tables

**Fig. 1:**
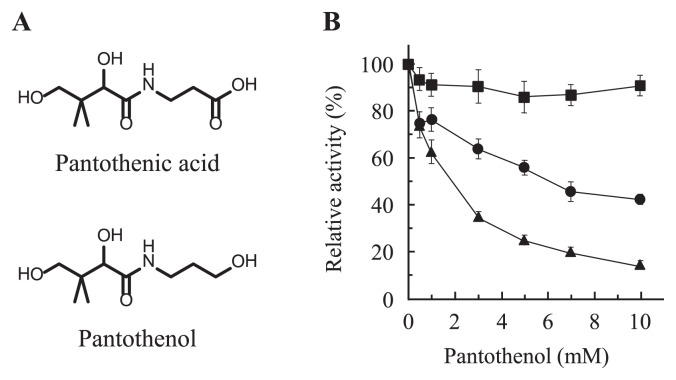
Inhibitory effect of pantothenol on three types of CoaAs. (A) Chemical structures of pantothenic acid and pantothenol. (B) Inhibition of *Ec*CoaA (circle), *Sa*CoaA (triangle), and *Pp*CoaA (square) activities by pantothenol. The assays were performed three times independently, and the results are indicated as the mean ± SD.

**Fig. 2:**
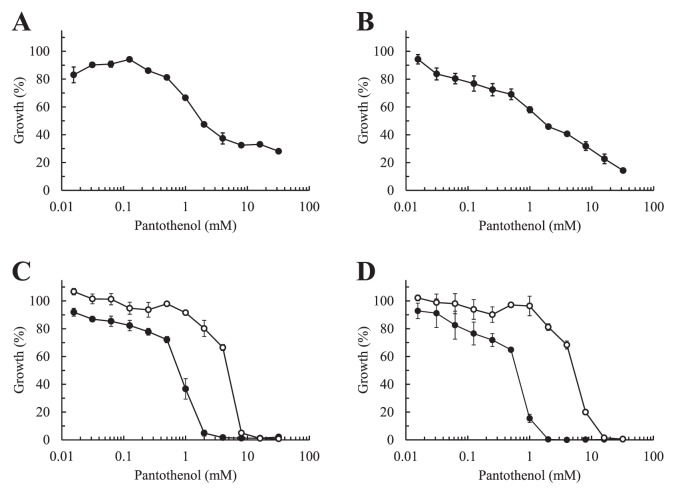
Inhibition of the growth of staphylococci by pantothenol. The inhibitory effect of pantothenol on the growth of *S. aureus* subsp. *aureus* NBRC 100910 (A), *S. epidermidis* NBRC 12993 (B), *S. epidermidis* NBRC 100911 (C), and *S. saprophyticus* subsp. *saprophyticus* NBRC 102446 (D) was examined in the presence (open circle) and absence (closed circle) of 1 μM pantothenate. The results are indicated as the mean ± SD (*n* = 3).

**Table 1:** Antimicrobial activity of pantothenol

Strain^a^	MIC (mM)	IC_50_ (mM)	Inhibition (%)^b^
Prokaryotic type I CoaA			
*Escherichia coli* K-12 substr. W3110 NBRC 12713	>32	na	11.6 ± 3.3
Prokaryotic type II CoaA			
*Staphylococcus aureus* subsp. *aureus* NBRC 100910	>32	1.82	71.9 ± 1.3
*S. epidermidis* NBRC 12993	>32	1.58	85.7 ± 0.8
*S. epidermidis* NBRC 100911	4	0.776	98.0 ± 0.5
*S. saprophyticus* subsp. *saprophyticus* NBRC 102446	2	0.641	99.7 ± 0.1
Prokaryotic type III CoaA			
*Pseudomonas putida* JCM 20089	>32	na	14.4 ± 0.3

na, not applicable.

a The strains listed in the table were obtained from the NBRC (NITE Biological Resource Center, Japan) and JCM (Japan Collection of Microorganisms).

b The values indicate the inhibition of growth in the presence of 32 mM pantothenol relative to growth without pantothenol (*n*=3, mean ± SD).
