# Supplementary material for: Antimicrobial Activity of Pantothenol against Staphylococci Possessing a Prokaryotic Type II Pantothenate Kinase
Source: Microbes Environ. 2014 Apr 22;29(2):224–6. doi: 10.1264/jsme2.ME13178 (PMC4103530; doi:10.1264/jsme2.ME13178)
Supplement: Supplementary file 1 [file 29_224_s1.pdf]

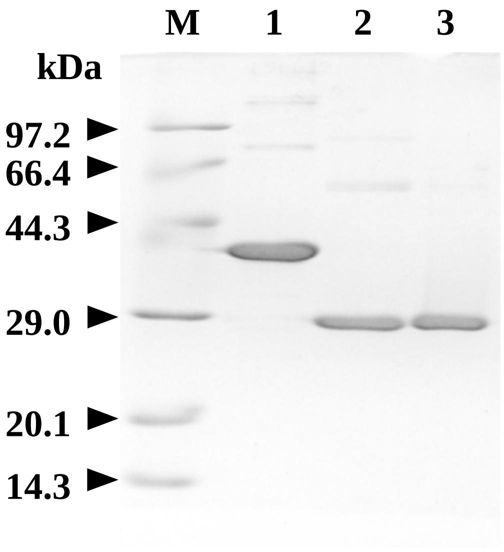

Fig. S1. SDS-12.5% polyacrylamide gel electrophoresis of purified recombinant pantothenate kinases (CoaAs). *E. coli* BL21(DE3) cells harboring pET15b/bPanK, pET-Sa-coaA, or pET-Pp-coaA were cultivated in 100 ml of LB medium with 50 µg ml^{-1} ampicillin or 25 µg ml^{-1} kanamycin at 30°C aerobically. When the turbidity at A_{600} reached ~0.6, IPTG was added to the culture broth at a concentration of 0.1 mM. After 6 h of cultivation, cells were collected by centrifugation, disrupted by sonication, and the supernatant was subjected to Ni-Sepharose resin. The His-tagged proteins were eluted with 200 mM imidazole for *E. coli* CoaA (*Ec*CoaA) and *S. aureus* CoaA (*Sa*CoaA) or with 50 mM EDTA for *P. putida* CoaA (*Pp*CoaA). Lane M, standard molecular mass markers; lane 1, 3 µg of the purified *Ec*CoaA; lane 2, 3 µg of the purified *Sa*CoaA; lane 3, 3 µg of the purified *Pp*CoaA. The numbers on the left indicate molecular masses. The theoretical molecular masses of the His-tagged *Ec*CoaA, *Sa*CoaA, and *Pp*CoaA are 38,523, 31,260, and 28,905, respectively.

Table S1. Purification of recombinant CoaAs

Purification step	Amt of protein[a] (mg)	Total activity[b] (nmol min^{-1})	Sp act (nmol min^{-1} mg^{-1})	Purification (fold)	Recovery (%)
*Ec*CoaA					
Crude extract	33.7	11,862	352	1	100
Ni-Sepharose	1.36	4,647	3,417	9.71	39.2
*Sa*CoaA					
Crude extract	27.2	16,401	603	1	100
Ni-Sepharose	7.08	12,015	1,697	2.81	73.3
*Pp*CoaA					
Crude extract	17.6	2,992	170	1	100
Ni-Sepharose	2.07	929	449	2.64	31.0

[a] Protein concentrations were measured by the Bradford method with bovine serum albumin as standard.

[b] The reaction mixture for *Ec*CoaA and *Sa*CoaA contained 91 μM D-[^{14}C]pantothenate (55 mCi $mmol^{-1}$), 2.5 mM ATP, 10 mM $MgCl_2$, 50 mM Tris-HCl (pH 7.5), and the enzyme solution in a total volume of 40 μL. For *Pp*CoaA, 60 mM NH_4Cl was added to the reaction mixture. The phosphorylation activity was measured at 30°C for 10 min and the reaction was stopped by addition of 4 μL of acetic acid. An aliquot of the mixture was deposited onto a Whatman DE81 ion exchange filter disk, and the filter disk was washed in three changes of 1% (v/v) acetic acid in 95% (v/v) ethanol. The produced 4'-phosphopantothenate on the disk was quantified by counting the dried disk in 3 mL of Ecoscint H.
